# Water-Drinking in Hot Weather

**Published:** 1871-02

**Authors:** 


WATER-DRINKING IN HOT WEATHER.—Drinking large quantities of cold water, when one is heated and thirsty, is injurious, and should not be allowed. But if people would use a little sense and less salt, butter, meat, fish, cake, and greasy food, they would not suffer so much with thirst, and there would be little occasion to complain of the water producing injury. Fruit, and the fresh unfermented juices of fruits, too, if more freely used in hot weather, would release the system from the need of so much water and promote health. We speak from knowledge on this subject, having observed the experiments with all sorts of drinks. Coffee is a favorite drink in the hay-field for many, but it destroys the appetite and corrupts the blood, in the end producing fever and weakness, and should not be used.—*Herald of Health.*

